# Antifungal Susceptibly Testing by Concentration Gradient Strip Etest Method for Fungal Isolates: A Review

**DOI:** 10.3390/jof5040108

**Published:** 2019-11-22

**Authors:** Eric Dannaoui, Ana Espinel-Ingroff

**Affiliations:** 1 Paris-Descartes University, Faculty of Medicine, 75006 Paris, France; 2 APHP, European Georges Pompidou Hospital, Parasitology-Mycology Unit, Microbiology Department, 75015 Paris, France; 3 Virginia Commonwealth University (VCU) Medical Center, Richmond, VA 23219, USA; victoria.ingroff@vcuhealth.org

**Keywords:** gradient strip method, Etest, antifungal susceptibility testing

## Abstract

Antifungal susceptibility testing is an important tool for managing patients with invasive fungal infections, as well as for epidemiological surveillance of emerging resistance. For routine testing in clinical microbiology laboratories, ready-to-use commercial methods are more practical than homemade reference techniques. Among commercially available methods, the concentration gradient Etest strip technique is widely used. It combines an agar-based diffusion method with a dilution method that determinates a minimal inhibitory concentration (MIC) in µg/mL. Many studies have evaluated the agreement between the gradient strip method and the reference methods for both yeasts and filamentous fungi. This agreement has been variable depending on the antifungal, the species, and the incubation time. It has also been shown that the gradient strip method could be a valuable alternative for detection of emerging resistance (non-wild-type isolates) as Etest epidemiological cutoff values have been recently defined for several drug-species combinations. Furthermore, the Etest could be useful for direct antifungal susceptibility testing on blood samples and basic research studies (e.g., the evaluation of the in vitro activity of antifungal combinations). This review summarizes the available data on the performance and potential use of the gradient strip method.

## 1. Introduction

Antifungal susceptibility testing (AFST) is now widely used and recommended for management of patients with invasive fungal infections such as candidiasis and aspergillosis [1,2,3]. AFST has also become an important tool for better epidemiological knowledge in rare fungal diseases [4,5,6,7]. There are currently broth microdilution reference techniques for both yeasts [8,9] and molds [10,11] that have been developed and standardized by the Clinical and Laboratory Standards Institute (CLSI) and by the European Committee for Antimicrobial Susceptibility Testing (EUCAST). Nevertheless, these reference techniques are time-consuming and more adapted for reference laboratories and large epidemiological surveillance studies. For routine testing in clinical microbiology laboratories, commercially available and ready-to-use methods may be a better alternative, as far as they are able to produce similar results with those obtained with the reference techniques. These methods may be based on different principles including microdilution broth (e.g., YeastOne) or agar diffusion (e.g., NeoSensitabs). 

## 2. Principle of the Concentration Gradient Strip (Etest) 

The concentration gradient strip technique is a combination of an agar-based diffusion method with a dilution method that determinates a minimal inhibitory concentration (MIC). A predefined exponential gradient of antifungal drug is immobilized on a plastic (Etest, bioMérieux, France) or impregnated on a paper (MTS, Liophilchem, Italy) strip. After homogenous inoculation of an agar plate, the strip is applied onto the agar surface and the drug is immediately released from the carrier to produce a continuous drug gradient in the agar medium. After incubation, an ellipse of growth inhibition is obtained, and the MIC is determined at the intersection of the ellipse with the scale on the upper side of the strip. The recommended medium used for testing is RPMI 1640 MOPS supplemented with 2% glucose and the incubation time is variable depending on the tested species [12].

## 3. Etest as a Routine AFST Method

### 3.1. Inhibition Patterns and Reading Problems

**Figure 1:**
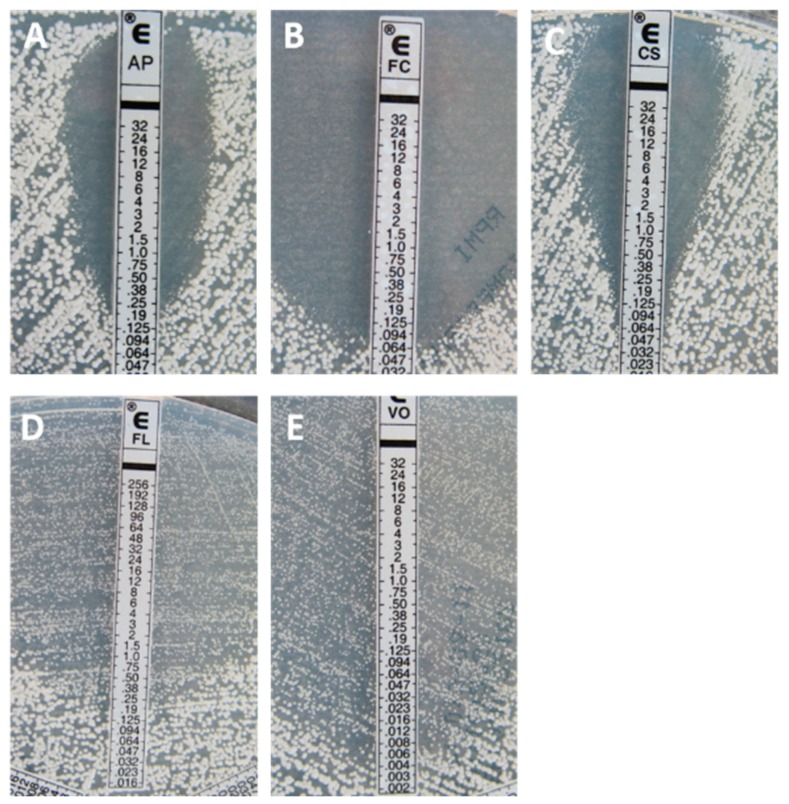
Typical inhibition pattern of amphotericin B (**A**), flucytosine (**B**), caspofungin (**C**), fluconazole (**D**), and voriconazole (**E**) against a wild-type (**WT**) isolate of *Candida albicans*.

**Figure 2:**
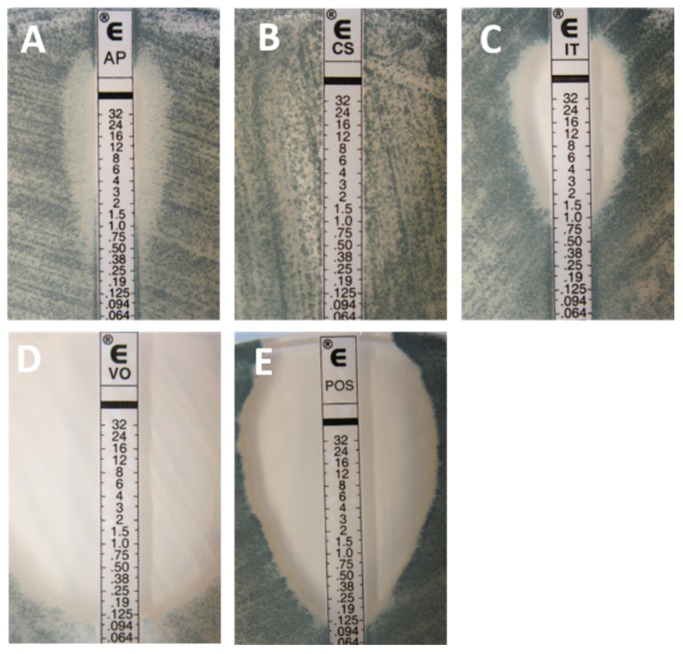
Typical inhibition pattern of amphotericin B (**A**), caspofungin (**B**), itraconazole (**C**), voriconazole (**D**), and posaconazole (**E**) tested against a wild-type (**WT**) isolate of *Aspergillus fumigatus*.

Inhibition patterns and reading endpoints (Figure 1 and Figure 2) are dependent on the drug and organism tested. Amphotericin B, in general, gives a sharp ellipse of inhibition that allows an easy MIC determination, since any colony inside the ellipse is important. The reading endpoint is a 100% inhibition for both yeasts and filamentous fungi [13,14]. Similar to most AFST methods, including the reference EUCAST and CLSI techniques, a trailing phenomenon [15] can be observed with the gradient strip method when azoles are tested against yeasts. With Etest, the trailing is visible as the presence of a lawn of microcolonies in the inhibition ellipse (Figure 1). The trailing is particularly important when testing specific species such as *C. albicans, C. glabrata,* and *C. tropicalis* and may lead to difficulties regarding the MIC endpoint. The reading endpoint for yeasts is an 80% inhibition. In contrast, a sharp and clear ellipse is generally observed when testing azoles against the filamentous fungi such as *Aspergillus* spp. (Figure 2), and therefore a 100% inhibition (trailing free ellipse) is the MIC endpoint [14]. For the echinocandins, the inhibition patterns are also different for both yeasts and filamentous fungi. When testing yeasts, a clear ellipse is generally seen which allows an easy MIC determination. The reading endpoint is an 80% inhibition (trailing within the ellipse). Nevertheless, two specific phenomena can be observed. The first one is the paradoxical effect corresponding to enhanced growth at high supra-MIC concentrations [16,17]. By the Etest method, it appears as the presence of microcolonies around the strip at the highest concentrations, within the inhibition ellipse [18]. The paradoxical effect is also observed using both CLSI and EUCAST reference microdilution broth techniques [19,20], but is not indicative of in vitro resistance or associated with therapeutic failure [21]. The second phenomenon is the dip effect, corresponding to a narrow inhibition zone at sub-MIC values, which can complicate the MIC determination [22]. When testing echinocandins against filamentous fungi, residual or trailing growth within the inhibition ellipse is generally observed and should be ignored for MIC determination [23]. The same phenomenon of partial inhibition is seen with broth microdilution techniques. Flucytosine is only tested against yeasts and, generally, gives large and clear ellipses. A 90% inhibition endpoint is used. It has been shown that testing flucytosine (on RPMI medium) against *Cryptococcus neoformans* can lead to erroneously high MICs [24,25,26]. 

### 3.2. Interlaboratory Reproducibility

Several studies have tested the interlaboratory reproducibility of Etest [26,27,28,29]. In one study, in which 83 strains of *Candida* spp. were tested in two laboratories [27], the overall reproducibility on RPMI, at +/−1 log_2_ dilution, was good for fluconazole (96%), ketoconazole (90%), flucytosine (84%), much lower for itraconazole (63%), and very poor for amphotericin B (4%). The same drugs have been evaluated in another study that tested two quality control strains in four laboratories [28]. The interlaboratory reproducibility at +/−1 log_2_ dilution was 99% to 100% for all five antifungals. In a large multicenter evaluation of reproducibility, 20 isolates (18 *Candida* spp. and 2 *C. neoformans*) were tested in 10 laboratories against amphotericin B, flucytosine, fluconazole, and itraconazole [26]. Overall, it was concluded that Etest is suitable to test amphotericin B and flucytosine against *Candida* spp. and less reliable for the azoles. In a more recent study, 198 isolates of *Candida* spp. were tested in four laboratories against amphotericin B and caspofungin [29]. The interlaboratory reproducibility, at +/−2 log_2_ dilutions, was very good at 97.5% and 97.1% for amphotericin B and caspofungin, respectively. 

### 3.3. Correlation with Reference Techniques

In most correlation studies, results of gradient strips are compared to results obtained by the CLSI or EUCAST microdilution broth reference techniques. The two main parameters used for comparison are the essential agreement (EA) and the categorical agreement (CA). The EA is the percentage of isolates for which MIC values by the gradient strip method are within +/−1 or +/−2 log_2_ dilutions of the values obtained by the reference method. In most of the studies, a threshold at +/−2 log_2_ dilutions is used. The CA is the percentage of isolates for which the same categorization (susceptible/intermediate/resistant) is obtained by the two methods. The availability of reference clinical breakpoints (CBPs) is a prerequisite for the calculation of CA percentages. Many problems are evident with most of these early categorizations and comparisons. Both triazole and echinocandin CPBs were adjusted to lower cutoffs as the molecular mechanisms of resistance were understood and more clinical data were available and also, different “resistant” amphotericin B cutoffs were used for these comparisons. Epidemiological cutoff values determined by the EUCAST (ECOFFs) or CLSI (ECVs) define the wild-type (WT) population and could be used for comparing results obtained by Etest and reference techniques. As the ECOFF and the ECV represent the same parameter, for simplification, the term ECV is used throughout this review. 

#### 3.3.1. Yeasts

Many studies have evaluated the agreement between gradient concentration strip methods and EUCAST/CLSI methods. Overall, a good level of agreement was generally found. 

##### Amphotericin B

Since the reference RPMI broth appeared to lack the ability to detect amphotericin B-resistant *Candida* isolates, other media such as the antibiotic medium 3 (AM3) were either compared to the CLSI data [30,31] or used instead of the RPMI agar in one of the comparisons with the EUCAST MICs (Table 1) [32]. However, in some instances the EA was not as good as that with the RPMI agar (Table 1) and lot-to-lot variation was also a problem when using the AM3 medium [33]. In one of the studies that evaluated the EA between the Etest and the CLSI method by using both AM3 and RPMI agars, results with the AM3 were lower than those with the RPMI agar for *C. parapsilosis* and *C. tropicalis* (overall EA 90% versus 97% with RPMI agar) [31]. Nevertheless, the overall EA between the CLSI and Etest RPMI agar was mostly >96% within the acceptable ±2 dilution range (1994 to 2011) for the most prevalent *Candida* species (*C. albicans*, *C. glabrata*, *C. parapsilosis*, and *C. tropicalis*) [30,31,34,35,36] and *C. neoformans* [37] (Table 1). The exceptions were the lower percentages of EA in one study where the Etest data were evaluated at both 24 and 48 h versus the CLSI method [38], for various species, including *C. lusitaniae* and *C. neoformans* [30,39]. The Etest was also evaluated for testing *C. krusei* with amphotericin B with an acceptable EA [30,34]. 

However, the comparison of Etest and EUCAST MICs yielded consistently and unusually low EA percentages, despite the fact that the Etest MICs were determined on both RPMI [32] and AM3 [25] agars (Table 1). 

Regarding rare *Candida* spp., one study provided Etest amphotericin B data for the emerging *C. auris*, as well as for the rare *C. haemulonii* and *C. pseudohaemulonii* on Mueller-Hinton (MH) agar supplemented with glucose and methylene blue as the “reference” method [36]. The comparison was against the Etest data on RPMI agar, other commercial assays and both reference methods (Table 1). All isolates were identified by sequencing the internal transcribed spacer (ITS) and D1/D2 regions of the 26S ribosomal DNA. The aim was to identify the best method for the detection of amphotericin B resistance. It was found that the Etest with MH yielded the widest amphotericin B MIC range and better “discriminated the susceptibility” of amphotericin B to these three species, 0.125–0.5 µg/ml for *C. auris,* and 4–32 µg/mL for the other two species. Blood isolates from three amphotericin B therapeutic failure patients were included in the study (MICs, 32 µg/mL) [36]. 

In summary, although good interlaboratory agreement has been observed for testing *Candida* spp. using Etest amphotericin B strips, data or isolates recovered after failure or during treatment are very scarce. 

##### Flucytosine

Flucytosine Etest data for yeasts were compared to those by the CLSI method in three studies [34,35,40] and with the EUCAST in two studies [25,32] (Table 2). The EA was excellent for all the *Candida* species tested, except in one study for *C. albicans* (EA 89%) [35] and in another one for *C. neoformans* (EA 70%) [40]. When both incubation times for the Etest results were evaluated [40], the 48 h Etest MICs consistently produced the higher EA percentages versus the CLSI macrodilution method for all the species evaluated, excluding *C. glabrata* (EA 100% with the 24 h Etest versus 83% with the 48 h results). It is noteworthy, as discussed below for the triazoles, that when testing fluconazole the first Etest reading yielded the highest EA for some species (Table 3). 

When Etest and EUCAST data were compared, contradictory EA results were reported again for all the *Candida* species tested (EA 62% to 82% versus 92% to 100%) [25,32]; the EA percentage for *C. neoformans* and flucytosine was unusually low (EA 35%) [25] (Table 2). 

##### The Triazoles

- Fluconazole

The published EA percentages between both CLSI and Etest fluconazole MICs for six species of *Candida* and *C. neoformans* [31,34,35,38,39,40,41,42,43] and the EUCAST [25,32] are presented in Table 3. Although the EA for fluconazole was >90% versus the Etest data for *Candida* spp. and *C. neoformans* in most studies, unacceptable EA percentages of <90% were also reported for each of the *Candida* spp. evaluated (Table 3) [34,35,38,40,41]. There are important caveats regarding these equivalences which are described next. The comparisons were mostly versus the CLSI macrodilution format. More important, some of these studies demonstrated the influence of the incubation time for the Etest data. For example, the overall percentages of EA were mostly higher at the Etest 24 h incubation times for *C. albicans* (82% to 97% versus 75% to 96%), *C. glabrata* (37% to 100% versus 34% to 83%) and *C. tropicalis* (56% to 100% versus 67% to 93%) [38,40,41]. However, for *C. parapsilosis*, the EA was best with the Etest 48 h MICs (89% to 100% versus 98% to 100%) [38,40]. These results could reflect the different growth rates, as well as the presence of higher amounts of trailing growth at the second incubation time. The fluconazole Etest was also evaluated using two other agar compositions (casitone an MH) against the CLSI; while the casitone agar yielded acceptable EA percentages, on the MH agar some EA values were below 90% [42]. 

The evaluation of the Etest against the EUCAST methodology once more provided disappointingly low percentages of agreement (Table 3) [25,32].

- Itraconazole, Posaconazole, and Voriconazole

The search of the literature provided less comparative data between the Etest and the CLSI or EUCAST methods for itraconazole [25,32,34,35,38], posaconazole [34,44,45], and voriconazole [25,32,34,37,38,39,43,46] (Table 3). We also found less information regarding the influence of the incubation time (24 versus 48 h) among the comparisons of the Etest with the CLSI method for these three triazoles [38,44]. Overall, the percentages of EA agreement were similar to those for fluconazole with both acceptable (>90%) and unacceptable (<90%) EA percentages. It is noteworthy that among the four most common *Candida* spp., the EA between the CLSI and Etest methods for *C. glabrata* was mostly below 90% for testing fluconazole and itraconazole [34,38,41,43]. The lowest EA values were among the evaluations for itraconazole, with similar and same percentages of EA at 24 and 48 h for the four most common *Candida* species, including *C. parapsilosis* [38] (Table 3). On the other hand, the EA indicated that posaconazole, and to a certain extent voriconazole, Etest MICs should be read at 24 h (higher or similar EA percentages at both incubation times) for *C. albicans*, *C. dubliniensis*, and *C. glabrata*. However, as for fluconazole, the Etest for *C. parapsilosis* should be read at 48 h (Table 3) [38,44]. Nevertheless, conclusions about the best incubation time are based on single studies and sometimes with a small number of isolates and species. It is noteworthy that the manufacturers advocate the first time reading, but that the confirmatory result should be at 48 h [12]. 

EA percentages between Etest and the CLSI microdilution method were acceptable at 48 h for *C. neoformans* versus fluconazole [39,40,42], posaconazole [44], and voriconazole [37,39]; some of those evaluations included large amounts of isolates and in some instances the first Etest incubation time reading provided the highest EA values (Table 3). 

Fewer studies evaluated the Etest for less prevalent species such as *C. dubliniensis* with posaconazole [44]; *C. guilliermondii* with fluconazole [43], posaconazole [44,45], and voriconazole [43,46]; *C. krusei* with fluconazole [34,40,42,43], itraconazole [34], posaconazole, and voriconazole [34,43,46]; and *C. lusitaniae* with fluconazole [34,40,42,43], itraconazole and posaconazole [34,44], and voriconazole [34,43] (Table 3). Overall, the EA agreement was >90% with a few exceptions for one or two triazoles. In addition, one study evaluated Etest data for 11 *C. kefyr*, 10 *C. rugosa*, eight *C. lipolytica* and eight *C. pelliculosa* resulting in EA agreement >90% with fluconazole and voriconazole (Table 3) [43].

Only two publications had EA data between the Etest and EUCAST methods for the triazoles [25,32]; the EA percentages were contradictory and disappointingly low with the following exceptions: fluconazole versus *C. albicans* and itraconazole and voriconazole versus *C. parapsilosis* (Table 3) [25,32]. 

##### The Echinocandins

Since the echinocandins were among the latest licensed agents to be evaluated for clinical use, with the exception of isavuconazole, fewer publications were found reporting EA percentages between the Etest and either the CLSI [34,47,48,49,50] or the EUCAST [25,51,52] methods (Table 4). Although all these studies were conducted with the CLSI microdilution format, the length of incubation was either 24 h (anidulafungin and micafungin) or 48 h (caspofungin). The EA for caspofungin Etest MICs against the CLSI method only produced consistently acceptable percentages in the three studies for *C. glabrata* and *C. krusei* [34,49,50]. The EA between the Etest and CLSI methods for anidulafungin was variable among the species, <90% values for all the species evaluated in either one of the two studies, with the exception of the EA for *C. tropicalis* (100%) (Table 4) [48,49]. However, the EA was >90% for all CLSI and Etest micafungin MIC pairs, except for the data for *C. parapsilosis* (87%) and *C. guilliermondii* (79%) (Table 4) [47,49]. Although the relationship between incubation time and EA percentages is not consistent, the impact of the incubation length is evident in several studies.

As for the other agents, only three evaluations were found in the literature on the EA between Etest and the EUCAST for the three echinocandins [25,51,52]. In the caspofungin study, the RPMI broth was replaced by the AM3 medium and the EA values were unacceptable for the species tested (<90%) [25]. In one of the two micafungin studies both 24 h and 48 h incubation times were evaluated; the pattern was similar to that discussed above for the triazoles, higher EA percentages for *C. parapsilosis* at 48 h and at 24 h for *C. albicans*, and *C. tropicalis* [52]. Therefore, it appears that as of now, the best Etest data for the echinocandins are those obtained for micafungin. 

##### Evaluation of EA According to MIC Ranges

As mentioned before, the EA was also reported as reference and Etest MIC ranges instead of the individual EA percentages for amphotericin B [29,53], flucytosine [53], fluconazole, voriconazole [53,54,55], and caspofungin [29,53,54]. The CLSI and Etest MIC ranges (microdilution and Etest MICs at 48 h in three of the four studies) were within the accepted <2 dilution difference for fluconazole [53,55], voriconazole, and flucytosine with the exception of *C. krusei* [53] and *C. tropicalis* [55]. However, for caspofungin and amphotericin B, CLSI and Etest MIC ranges appeared to be >2 dilutions for most of the species tested [29,53,54]. These discrepancies reflected the reported overall agreement for caspofungin from 77% to 97% [29,53,54] and for amphotericin B >89% [29,53]. When both incubation times were evaluated in a single study [54], the highest EA percentages were obtained for the first MIC readings by both methods (overall EA 98% for voriconazole and 100% for caspofungin). Other important observations also were reported in one of four studies [55], i.e., the presence of double growth zones within the whole Etest ellipse when testing some isolates of *C. albicans* and *C. glabrata* with fluconazole, as well as a substantial trailing among *C. tropicalis* and *C. glabrata* isolates. This heavy trailing precluded the definition of Etest fluconazole MICs for a substantial number of isolates of these two species [55]. 

Although results were similar for the comparison of the Etest and EUCAST methods for voriconazole, the incubation time did not influence the comparison for caspofungin MICs (94% and 95%) [54].

#### 3.3.2. Filamentous Fungi

Correlation between gradient strip and reference microdilution methods for filamentous fungi has been mostly evaluated for *Aspergillus* spp. [23,44,56,57,58,59,60,61,62,63,64,65,66,67,68,69,70,71,72,73,74,75,76,77,78], *Fusarium* spp. [60,63,65,66,68,73,76,79,80,81,82], *Scedosporium* spp. [60,63,66,73,76,83], and Mucorales species [44,63,65,66,73,76,81,84,85,86]. 

##### *Aspergillus* spp. 

For *Aspergillus* spp, more than 25 comparative studies have been performed (Table 5), in which more than 3000 isolates were tested against different drugs.

**Figure 3:**
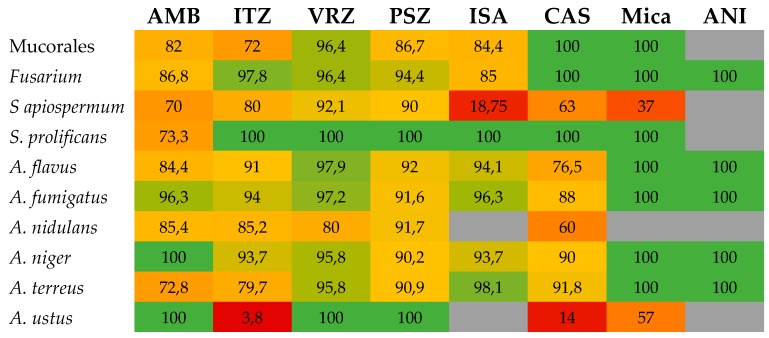
Heatmap showing the level of essential agreement (EA) between Etest and microdilution broth reference techniques for the different drug-bug combinations for filamentous fungi. When several studies were available, a mean EA was calculated. Numbers represent the percentage of EA and boxes are colored from red (low EA) to green (high EA). AMB, amphotericin B; CAS, caspofungin; ISA, isavuconazole; ITZ, itraconazole; Mica, micafungin; PSZ, posaconazole; VRZ, voriconazole; ANI, anidulafungin.

For amphotericin B, the overall EA between Etest and reference techniques was mostly between 80% and 100% (Table 5 and Figure 3). Most of the studies showed a better EA when the Etest was read at 24 hours [44,60,61,67,69,71]. This may be due to the higher amphotericin B MICs by the Etest when the incubation time is extended to 48 hours, while similar MICs were noted among CLSI MICs at both incubation times. Almost all studies showed that overall, higher amphotericin B MICs were obtained by Etest than those obtained by the CLSI method [44,60,61,66,67,69,71,76]. This was more pronounced for *A. flavus* and *A. terreus* and could explain the lowest EA percentages for these two species [60,61,66,69,76]. Indeed, major discrepancies have been reported in some studies for *A. terreus* and *A. flavus* with EA of 16% and 40%, respectively [66,69] and more recently of 79.7% [78]. 

The correlation between azole Etest and mostly CLSI MICs for *Aspergillus* spp. has been reported for itraconazole, voriconazole, posaconazole, and isavuconazole. Most EA have been >90% (Table 5 and Figure 3), but lower percentages (64% to 88%) have also been noted [65,66,67,69,71,72]; the lowest EA (64%) was for the combination of posaconazole and *A. terreus* [66]. As for amphotericin B MICs, Etest itraconazole MICs were usually higher than CLSI values [60,65,67,69,76,78,87]. In contrast, Etest voriconazole, posaconazole, and isavuconazole MICs were generally lower than CLSI endpoints [57,58,61,63,66,72,74,87]. 

Echinocandin MICs by the Etest have been evaluated in several studies, mainly for caspofungin [23,62,66,68,74,78] and to a lesser extent for micafungin [66,68,78] and anidulafungin [68,74,78]. EA was generally good (up to 100%), but lower values also have been reported in some of the studies (Table 5). It must be noted that for some specific species such as *A. ustus*, performance of Etest was much lower with EAs of 57% and 14% for micafungin and caspofungin, respectively [66]. 

Overall, Etest is an alternative to reference methods for testing *Aspergillus* spp., particularly for *A. fumigatus* versus the triazoles, since Etest results are usually above the ECV for mutant strains (non-WT). Although a good correlation between results obtained by Etest and by reference techniques are reported, the recent ESCMID-ECMM guidelines on diagnosis and management of *Aspergillus* diseases only marginally support the use of Etest for antifungal susceptibility testing of *Aspergillus* clinical isolates [3].

##### Mucorales

Various studies have compared the performance of the Etest with CLSI or EUCAST methods for the antifungal susceptibility testing of the Mucorales (Table 6), mostly for amphotericin B, itraconazole, and posaconazole. Moderate to good percentages of EA were generally observed. Although the EA was >90% in several studies for amphotericin B [44,66,76], the range was between 70% to 80% in other reports [81,84,86]. For posaconazole, the EA ranged from 77% to 100% [44,65,66,81,84,85,86]. A CA of 67% and 87% (by using CBPs of ≥1 µg/ml) were reported for amphotericin B and posaconazole, respectively [85]. For itraconazole, the EA was 50% to 83% [65,73,76,86], while an acceptable EA of 84% was observed during the only study that evaluated the Etest for testing isavuconazole against 45 isolates of Mucorales (62). Some studies have evaluated voriconazole, micafungin, and caspofungin with, not surprisingly, good agreement of 90% to 100% for these agents devoid of activity against Mucorales [65,66]. Therefore, the only acceptable EA was for testing amphotericin B by the Etest and the Mucorales.

##### *Fusarium* spp.

Antifungal susceptibility testing of *Fusarium* spp. is now recommended in patient care [88]. The comparisons of Etest results for *Fusarium* spp. to those obtained by the CLSI or EUCAST are depicted in Table 7. Three studies evaluated amphotericin B Etest strips for *Fusarium* isolates, the EA was either >90% [66,79,81,82] or lower [60,76,80]. In another study, the Etest was compared with both reference methods and the EA was either 95% and 90% against EUCAST and CLSI MICs, respectively [79]. In the latter study, the CA (based on a 4 µg/ml ECV for *Fusarium* spp.) was 100% with the EUCAST, but 85% with the CLSI MICs [79]. Among the azoles, the EA have ranged from 80% to 100% in most studies for itraconazole, voriconazole, and posaconazole [60,65,73,76,79,80,82]. A high level of CA between Etest voriconazole (90%, based on a 4 µg/mL ECV) and posaconazole (95%, based on a 2 µg/mL ECV) and both CLSI and EUCAST MICs have also been reported [79]. The only study that evaluated Etest isavuconazole strips for *Fusarium* spp. (20 isolates) reported an 85% EA with CLSI MICs [63]. When testing the echinocandins, which exhibited no in vitro activity against *Fusarium* spp., a 100% agreement was found between Etest caspofungin, micafungin, and anidulafungin and CLSI MICs [66,68,74]. Overall, the Etest could to be a good alternative method for testing of *Fusarium* spp., but unusually high MICs should be confirmed by the CLSI method since ECVs for some of these species only are available by this method.

##### *Scedosporium* 

Some studies also have evaluated the agreement between Etest and CLSI MICs for *Scedosporium* isolates (Table 8) [60,63,66,73,76,83]. Overall, the agreement was dependent on the antifungal and the species tested (i.e., *S. apiospermum* vs. *S. prolificans*). For amphotericin B, an EA of 80% to 100% has been reported in two studies [60,66] with an unacceptable lower agreement (20%) elsewhere due to higher Etest MICs [76]. Methodological differences may account for these troublesome discrepancies, especially the incubation time. Among the azoles, the EA was >90% between Etest itraconazole, voriconazole, and posaconazole and CLSI MICs in four studies [60,66,73,83], but 50% when itraconazole MICs for *S. apiospermum* were evaluated [76]. Isavuconazole strips only have been evaluated in one study where the EA was 100% for *S. prolificans,* but unacceptably lower (18.7%) for *S. apiospermum* [63]. For the echinocandins, the EA between Etest and CLSI MICs was better for *S. prolificans* (100% for both caspofungin and micafungin) than for *S. apiospermum* (63% for caspofungin and 37% for micafungin) [66]. Although the Etest could be a valuable alternative, there is a need to further evaluate the optimal incubation times for isolates of *S. prolificans* and *S. apiospermum* and the various agents. 

### 3.4. Ability to Detect Acquired Resistance

An important point is to know if the gradient strip Etest method can correctly detect isolates with decreased susceptibility to antifungal agents. CBPs are not available for any commercial method but ECVs have been defined for the Etest and SYO methods for some *Candida* and *Aspergillus* species [89]. Therefore, we cannot talk about resistance by any of the commercial methods. However, the ability to identify isolates, as either mutants (non-WT) or WT, can be evaluated using method specific ECVs. This ability to detect non-WT isolates has been thoroughly reviewed recently for testing some yeast species against amphotericin B and the echinocandins for prevalent *Candida* spp(Espinel-A and Dannaoui E. Under preparation) . 

Briefly, for amphotericin B, it has been shown in several studies that Etest was able to better detect the decreased susceptibility of some yeasts than reference methods [30,31,36,90,91,92,93]. Etest was better than the CLSI in recognizing non-WT isolates among *C. neoformans* [92] and *Candida lusitaniae* [90,93]. For the filamentous fungi, one study compared Etest and CLSI methods for some *A. flavus* isolates [94] and the Etest results better correlated with the data from an experimental model of systemic aspergillosis. 

For echinocandins, studies have compared the Etest to either the CLSI or EUCAST methods for detecting non-WT *Candida* isolates [49,89,95,96,97,98,99]. Overall, Etest reliably detected *Candida* fks mutants and in some studies better discriminated between WT and mutant isolates than the reference techniques [95,96]. In addition, in the largest study that comprised data from 140 molecularly defined echinocandin mutants of *Candida* spp., anidulafungin Etest classified 92% of the *Candida* fks mutants as non-WT, while the detection was lower for caspofungin (75%) and micafungin (84%) [89].

For the triazoles, itraconazole, voriconazole, posaconazole, and isavuconazole, Etest MICs have been evaluated for the detection of *A. fumigatus* mutants (CYP51A substitutions associated with azole resistance) [57,100,101,102,103]. Globally, it have shown that while the Etest itraconazole ECV for this species was a good detector [101], it was not as efficient as the CLSI method for testing posaconazole [103]. In summary, in some reports, the Etest was better in detecting the potentially itraconazole resistant *A. fumigatus* isolates as well better discriminated between amphotericin B MICs for some *Candida* spp. and *C. neoformans*.

### 3.5. Etest for Direct Antifungal Susceptibility Testing on Blood Samples 

When a blood culture is positive, it is necessary to subculture the strain on agar medium for performing antifungal susceptibility testing. This step implies a 24 h to 48 h further delay for reporting the susceptibility results. For this reason, the performance of antifungal susceptibility testing directly from blood culture bottles without that extra step was evaluated. 

The Etest method for direct MIC determination from positive blood culture bottles was evaluated and described below [104,105,106,107,108]. The first evaluation was conducted on 138 positive blood cultures containing yeasts (mainly *Candida* spp.). The agreement was acceptable (81.8% to 89.4%) against the CLSI method for testing amphotericin B, flucytosine, fluconazole, and ketoconazole, but not for itraconazole (69.7% [105]). In another study, direct Etest was again compared to the CLSI microdilution method on 328 blood culture samples (195 collected and 133 laboratory prepared); the direct test performance was good with a low rate of false susceptibility detection (i.e., very major errors) for fluconazole, voriconazole, isavuconazole, and caspofungin [107]. Lower agreement was found for amphotericin B and posaconazole. In a prospective study that compared Etest to the CLSI disk diffusion method, a CA of 100% for fluconazole, voriconazole, amphotericin B and 86.2% for caspofungin was reported between the two techniques [108]. Two other studies focused on the ability of direct Etest to detect resistance to fluconazole and echinocandins in blood cultures [104,106]. In the first study, the Etest was evaluated on blood cultures for the detection of fluconazole resistance in *C. albicans*. The results showed 100% CA with reference broth microdilution techniques (both CLSI and EUCAST) when trailing was ruled out [106]. In the second study, direct susceptibility by Etest was performed for micafungin and anidulafungin-susceptible and -resistant strains. Overall, both the EA and CA between direct Etest and conventional EUCAST were >97% [104].

In summary, the direct Etest could be a reliable method for antifungal susceptibility testing of blood yeasts isolates for the faster detection of both fluconazole and echinocandin resistance in some *Candida* isolates.

### 3.6. Etest Specific ECVs

Because ECVs are method dependent, several studies have gathered and analyzed results from multiple and independent laboratories to define Etest-specific ECVs [89,101,109]. Etest-specific ECVs are currently available for several species of *Candida* and *Aspergillus* (Table 9 and Table 10).

## 4. Etest as an AFST Research Tool

As the gradient concentration strip method is easy-to-use and mostly reproducible, it has been widely used in the research area for different purposes. In some instances, it has been used for isolation of flucytosine-resistance progeny in *C. tropicalis* [110] or for testing fluconazole susceptibility of laboratory *C. albicans* mutants [111,112]. Nevertheless, one of the most important research applications of Etest has been the in vitro evaluation of antifungal combinations against a wide range of fungal species.

### Combination Studies

The activity of antifungal combinations is most commonly evaluated by the checkerboard method, performed following the guidelines of the broth microdilution CLSI or EUCAST techniques. Time-kill curves are also used to evaluate the combined fungicidal activity of antifungal agents. Nevertheless, these techniques are time-consuming. In a comparison of several such techniques (Etest, checkerboard, and time-kill), the Etest was simple to use, time-efficient, reproducible, and was proposed as an alternative method [113]. Moreover, because the Etest is an agar diffusion assay, it can also be used to support or confirm results of antifungal interactions detected by checkerboard or time-kill methods [114,115,116,117,118,119,120,121]. Different protocols can be used for testing antifungal combination of a drug A with a drug B by the Etest. The first method is mainly used when one of the partner drugs is not an antifungal agent or when there is no available Etest for this drug. The MIC of drug A is determined by Etest either alone or after drug B has been included in the agar at a fixed concentration [114,115,118,122,123,124,125,126,127]. The second method is used when Etest are available for both drug A and drug B. In this case, after the determination of the MICs for both drugs alone, the combination can be evaluated by the following three main protocols: (i) The fixed ratio protocol where the strip of drug A is applied on the agar for 1 hour, the strip is removed, and the strip of drug B is applied exactly on the same position [113,128]; (ii) the MIC/MIC ratio where the strip of drug A is applied on the agar for 1 hour, removed, and the strip of drug B is applied after vertical transposition such as MIC_A_ falls on MIC_B_; and (iii) the cross protocol where the strips of A and B are crossed at 90° angle at the position of their respective MIC [129]. 

Etest based strategies have mainly been used to evaluate antifungal combinations against *Candida* spp. [114,116,117,118,119,123,124,127,130], *Aspergillus* spp. [120,121,122,123,124,125,126,131], and Mucorales [115].

In *C. glabrata*, the combination of caspofungin with amphotericin B, showed synergy in 40% of the cases and a good concordance of 92% between Etest and time-kill studies was found [116]. For the combination of caspofungin with azoles (fluconazole, itraconazole, and voriconazole), mainly indifference was found with a concordance of 66% to 86% with the time-kill method [117]. In another study [114], the combination of terbinafine with fluconazole or voriconazole against *Candida* spp. showed a good concordance of the Etest results with those of the checkerboard method and it was concluded that Etest is a suitable method to determine drug interactions. Etest has also been used to test combination of antifungals with non-antifungal drugs against *Candida* spp [118,119,130]. A synergistic interaction was found between polymyxin B and fluconazole against *C. glabrata* by time-kill and Etest with a concordance of 60% [119]. In another study that evaluated the combination of polymyxin B with caspofungin, more synergistic interactions were found with Etest than with time-kill [130]. In one study, the interaction of amphotericin B with flucytosine against *Cryptococcus neoformans* was tested by checkerboard, time-kill, and Etest. Although some synergy was found by checkerboard and time-kill, indifferent interaction was found for all strains by Etest [132]. This lack of concordance between Etest and the other techniques was probably due to the known problems of testing flucytosine against *C. neoformans* by Etest [24].

In *Aspergillus* spp., several studies have reported the used of Etest for testing combination of azoles with echinocandins [120,121,131]. In the first two studies, the combination of voriconazole with anidulafungin and the combination of isavuconazole with the three echinocandins against azole-susceptible and -resistant *Aspergillus* species showed mainly indifferent interactions by Etest. Similar results were obtained by checkerboard, demonstrating a good concordance between the two techniques. In another study, Etest and checkerboard were compared to test combination of azoles with echinocandins against itraconazole-resistant isolates of *A. fumigatus* [131]. Overall, the results showed variable concordance depending on the combination. Etest was also used against *A. fumigatus* to visualize antagonism of voriconazole with flucytosine that was initially observed by checkerboard [125].

This Etest strategy has also been evaluated against individual strains to visualize the mode of interaction of antifungal with non-antifungal drugs (incorporated in the agar) against both *Candida* and *Aspergillus* [123,124,127]. In that way, it has been shown that radicicol, an inhibitor of hsp90, and cyclosporin A could synergize the activity of caspofungin against *A. terreus*, and the activity of azoles against *C. albicans* [123]. The same synergistic interaction between geldanamycin and fluconazole against *C. albicans*, and between geldanamycin and caspofungin against *A. fumigatus*, was also demonstrated by a similar approach [124]. 

In Mucorales, one study evaluated the interaction of cyclosporin A with either amphotericin B or posaconazole and showed synergy both by Etest and by checkerboard [115].

## 5. Conclusions and Perspectives

The Etest gradient strip method is a valuable alternative to the reference techniques for routine antifungal susceptibility testing in clinical laboratories with some caveats. The optimal incubation time needs to be clarified as this review has provided some insights that this testing condition could be antifungal, agent, and species dependent. For its application for drug-bug combination studies, both the incubation and the appropriate reading endpoints should be further explored. The usefulness of Etest ECVs for the detection of emerging resistance (non-WT isolates) has only been evaluated for some antifungal agent and species combinations, i.e., amphotericin B (for certain *Candida* spp.), echinocandins (for prevalent *Candida* spp.), and triazoles (mostly for *A. fumigatus*). The interlaboratory modal reproducibility of other agent and species combinations need additional evaluation, e.g., the triazoles versus *Candida* spp. Therefore, further studies are warranted for improving its routine use as a detector of non-WT or emerging resistance, the most important role of any susceptibility method. It is also necessary to determine the degree of correlation with reference techniques or the reproducibility of Etest MICs for less prevalent *Candida* and filamentous fungal species. 

## Figures and Tables

**Table 1:** Percent essential agreement (EA) (+/− two dilutions) of Etest and reference amphotericin B minimal inhibitory concentrations (MICs).

Species	CLSI and Etest					EUCAST and Etest		
	No. Isol	EA%	Comments	EA % AM3	Ref	No. Isol	EA%	Ref
*C. albicans*	53	96	48 h macro Etest	98	[31]	345 ^d^	81	[25]
	266	98	48 h micro Etest		[30]	54	15	[32]
	94	99	48 h micro		[34]			
	123	97	48 h macro Etest		[35]			
	181	84/88	micro Etest 24/48 h		[38]			
*C. glabrata*	9	100	48 h macro Etest	100	[31]	104 ^d^	69	[25]
	102	100	48 h micro Etest		[30]	11	44	[32]
	38	100	48 h micro		[34]			
	38	77/86	micro Etest 24/48 h		[38]			
*C. krusei*	28	96	48 h micro Etest		[30]			
	5	100	48 h micro		[34]			
*C. parapsilosis*	10	100	48 h Macro/Etest	90	[31]	68 ^d^	65	[25]
	142	100	48 h micro Etest		[30]	38	2,7	[32]
	31	100	48 h Micro		[34]			
	47	85/85	micro Etest 24/48 h		[38]			
*C. tropicalis*	79	99	48 h micro Etest		[30]	54 ^d^	61	[25]
	34	100	48 h micro		[34]	33	22	[32]
	13	92	48 h macro Etest	85	[31]			
	48	80/89	micro Etest 24/48 h		[38]			
*C. lusitaniae*	19 ^a^	89	48 h micro Etest		[30]			
	8	100	48 h micro		[34]			
*C. auris*	20	100	24 h micro Etest ^b^		[36]	20	100	[36]
*C. neoformans*	162	99	micro Etest 72 h		[37]	26 ^d^	73	[25]
	85	83	micro Etest 48–72 h		[39]			

^a^, Including two resistant *C. lusitaniae* isolates. Etest for some isolates on AM3, MH, and casitone agars [30]. ^b^, MH medium used as the "Etest reference"; rare *C. haemulonii* and *C. pseudohaemulonii* also evaluated [36]. ^c^, RPMI with 2% dextrose [39]. ^d^, Etest MICs on AM3 agar [25].

**Table 2:** Essential agreement (EA) (+/− two dilutions) of flucytosine Etest and reference MICs.

Species	CLSI and Etest				EUCAST and Etest		
	No. Isol	EA%	Comments	Ref.	No. Isol	EA%	Ref
*C. albicans*	28	93/100 ^a^	macro 24/48 h Etest	[40]	166	96	[25]
	94	93	48 h micro	[34]	54	72	[32]
	123	89 ^b^	48 h macro Etest	[35]			
*C. glabrata*	6	100/83	macro 24/48 h Etest	[40]	46	100	[25]
	38	100	48 h micro	[34]	11	78	[32]
*C. krusei*	7	86/100	macro 24/48 h Etest	[40]			
	5	100	48 h micro	[34]			
*C. parapsilosis*	7	86/100	macro 24/48 h Etest	[40]	26	92	[25]
	31	100	48 h micro	[34]	38	82	[32]
*C. tropicalis*	14	93/100	macro 24/48 h Etest	[40]	25	100	[25]
	34	94	48 h micro	[34]	33	62	[32]
*C. lusitaniae*	6	83/100	macro 24/48 h Etest	[40]			
	8	100	48 h micro	[34]			
*C. neoformans*	10	70/60	macro 24/48 h Etest	[40]	20	35	[25]

^a^, highest EA % at the second Etest reading [40] and ^b^, data given for other species and non-*C. albicans* [35].

**Table 3:** Percentage essential agreement (EA) (+/− two dilutions) of azoles (fluconazole, itraconazole, posaconazole, and voriconazole) Etest and reference MICs.

Species/Agent	CLSI and Etest						EUCAST and Etest		
	No. Isol	EA%	Comments	EA % M-H Agar	EA % Casitone	Ref	No. Isol	EA %	Ref
**Fluconazole**									
*C. albicans*	28	82/75 ^a^	macro Etest 24/48 h			[40]	354	67	[25]
	122	93/79	macro Etest 24/48 h			[41]	54	91	[32]
	208	97/96	micro Etest 24/48 h			[38]			
	23	96	macro Etest 48 h			[31]			
	161	90	micro Etest 48 h	50	95	[42]			
	123	85	48 h macro Etest			[35]			
	94	98	48 h micro			[34]			
*C. glabrata*	12	92	macro Etest 48 h			[31]	110	73	[25]
	41	100	micro Etest 48 h	28	95	[42]	11	82	[32]
	6	100/83 ^a^	macro Etest 24/48 h			[40]			
	34	77/75	micro Etest 24/48 h			[38]			
	41	37/34	macro Etest 24/48 h			[41]			
	38	82	48 h micro			[34]			
*C. guilliermondii*	53 ^b^	91	micro Etest 48 h			[43]			
	7	100/100 ^a^	macro Etest 24/48 h			[40]			
*C. krusei*	32	100	micro Etest 48 h	90	97	[42]			
	118	97	micro Etest 48 h			[43]			
	5	40	48 h micro			[34]			
*C. parapsilosis*	7	100/100 ^a^	macro Etest 24/48 h			[40]	69	78	[25]
	12	100	macro Etest 48 h			[31]	38	82	[32]
	29	100	micro Etest 48 h	17	100	[42]			
	54	89/98	micro Etest 24/48 h			[38]			
	31	87	48 h micro			[34]			
*C. tropicalis*	14	100/93 ^a^	macro Etest 24/48 h			[40]	54	85	[25]
	13	93	macro Etest 48 h			[31]	33	58	[32]
	35	91	micro Etest 48 h	91	100	[42]			
	34	100	48 h micro			[34]			
	52	96/85	micro Etest 24/48 h			[38]			
	45	56/67	macro; 24/48 h Etest			[41]			
*C. lusitaniae*	31	97	micro Etest 48 h	45	100	[42]			
	8	100	48 h micro			[34]			
	56	100	micro Etest 48 h			[43]			
	6	83/83 ^a^	macro Etest 24/48 h			[40]			
*C. neoformans*	40	97	micro Etest 48 h	87	93	[42]	30	77	[25]
	97	95 ^c^	micro Etest 48–72 h			[39]			
	10	90/83 ^a^	macro Etest 48/72 h			[40]			
**Itraconazole**									
*C. albicans*	94	97	48 h micro			[34]	109	73	[25]
	123	72	48 h macro Etest			[39]	54	72	[32]
	205	96/95	micro Etest 24/48 h			[38]			
*C. glabrata*	38	89	48 h micro			[34]	31	68	[25]
	34	75/77	micro Etest 24/48 h			[38]	11	55	[32]
*C. krusei*	5	80	48 h micro			[34]			
*C. lusitaniae*	8	100	48 h micro			[34]			
*C. parapsilosis*	31	100	48h micro			[34]	13	92	[25]
	52	95/95	micro Etest 24/48 h			[38]	38	95	[32]
*C. tropicalis*	34	94	48 h micro			[34]	14	50	[25]
	46	85/85	micro Etest 24/48 h			[38]	33	73	[32]
*C. neoformans*	NA					NA	11	72	[25]
**Posaconazole**									
*C. albicans*	25	92/92	micro Etest 24/48 h			[44]			
	174	98	micro Etest at 48 h			[45]			
	94	100	48 h micro			[34]			
*C. dubliniensis*	10	92/92	micro Etest at 24/48 h			[44]			
*C. glabrata*	57	93	micro Etest at 48 h			[45]			
	10	100/90	micro Etest at 24/48 h			[44]			
	38	95	48 h micro			[34]			
*C. krusei*	5	100	micro Etest at 48 h			[45]			
	10	100/100	micro Etest at 24/48 h			[44]			
	5	100	48 h micro			[34]			
*C. lusitaniae*	10	70/90	micro Etest at 24/48 h			[44]			
	8	100							
*C. parapsilosis*	39	85	micro Etest at 48 h			[45]			
	10	60/90	microm Etest at 24/48 h			[44]			
	31	100	48 h micro			[34]			
*C. tropicalis*	31	97	micro Etest at 48 h			[45]			
	10	70/100	micro Etest at 24/48 h			[44]			
	34	100	48 h micro			[34]			
*C. guilliermondii*	6	88	micro Etest at 48 h			[45]			
	10	80/100	micro Etest at 24/48 h			[44]			
*C. neoformans*	15	93	micro Etest at 72 h			[44]			
**Voriconazole**									
*C. albicans*	174	99 ^d^	micro Etest 48 h			[46]	308	88	[25]
	94	93	48 h micro			[34]	54	87	[32]
	212	99/96	micro Etest 24/48 h			[38]			
*C. glabrata*	55	91 ^d^	micro Etest 48 h			[46]	98	82	[25]
	38	89	48 h micro			[34]	11	60	[32]
	44	93/100	micro Etest 24/48 h			[38]			
*C. krusei*	5	100 ^d^	micro Etest 48 h			[46]			
	5	100	48 h micro			[34]			
	118	99	micro Etest 48 h			[43]			
*C. lusitaniae*	8	100	48 h micro			[34]			
	56	100	micro Etest 48 h			[43]			
*C. parapsilosis*	39	100 ^d^	micro Etest 48 h			[46]	59	86	[25]
	31	97	48 h micro			[34]	38	95	[32]
	55	96/100	micro Etest 24/48 h			[38]			
*C. tropicalis*	31	100 ^d^	micro Etest 48 h			[46]	48	81	[25]
	34	85	48 h micro			[34]	33	73	[32]
	54	100/91	micro Etest 24/48 h			[38]			
*C. guilliermondii*	6	100 ^d^	micro Etest 48 h			[46]			
	53 ^b^	79	micro Etest 48 h			[43]			
*C. neoformans*	93	91 ^c^	micro Etest 48–72 h			[39]	22	86	[25]
	162	94	micro Etest 72 h			[37]			

^a^, overall, highest % at the first Etest reading, especially for *C. albicans*, *C. glabrata,* and *C. neoformans* [40]. ^b^, also data for 11 *C. kefyr*, 10 *C. rugosa*, 8 *C. lipolytica* and *C. pelliculosa,* and 7 *C. dubliniensis* [43]. ^c^, RPMI with 2% dextrose for the CLSI method [39]. ^d^, provided data using AM3 and casitone agar (lower % of agreement) [46].

**Table 4:** Essential agreement (EA) (+/− two dilutions) of echinocandins (caspofungin, micafungin, and anidulafungin) Etest and reference MICs.

Species/Agent	CLSI and Etest						EUCAST and Etest			
	No. Isol	EA%	Comments	EA% AM3	EA% Casitone	Ref CLSI	No. Isol	EA%	Comments	Ref
**Caspofungin**										
*C. albicans*	486	95	Micro/Etest 48h	88	85	[50]	120	80		[25]
	94	89	48 h Micro			[34]				
	32	94	Micro/Etest 24h			[49]				
*C. glabrata*	96	99	Micro/Etest 48h	100	100	[50]	45	67		[25]
	38	90	48 h Micro			[34]				
	34	94	Micro/Etest 24h			[49]				
*C. krusei*	11	100	Micro/Etest 48h	100	80	[50]				
	5	100	48 h Micro			[34]				
	11	91	Micro/Etest 24h			[49]				
*C. lusitaniae*	8	100	48 h Micro			[34]				
*C. parapsilosis*	47	79	Micro/Etest 48h	49	77	[50]	29	90		[25]
	31	100	48 h Micro			[34]				
	25	100	Micro/Etest 24h			[49]				
*C. tropicalis*	51	86	Micro/Etest 48h	23	40	[50]	20	60		[25]
	34	88	48 h Micro			[34]				
	12	100	Micro/Etest 24h			[49]				
*C. guilliermondii*	33	100	Micro/Etest 48h	100	83	[50]				
	19	84	Micro/Etest 24h			[49]				
*C. neoformans*							4	100		[25]
**Micafungin**										
*C. albicans*	125	97	Micro/Etest 24h			[47]	31	93/90	24/48 h	[52]
	32	100	Micro/Etest 24h			[49]	159	100		[51]
*C. glabrata*	52	100	Micro/Etest 24h			[47]	40	89/90	24/48 h	[52]
	34	94	Micro/Etest 24h			[49]	152	99		[51]
*C. krusei*	39	95	Micro/Etest 24h			[47]	10	80/80	24/48 h	[52]
	11	100	Micro/Etest 24h			[49]	127	98		[51]
*C. parapsilosis*	31	87	Micro/Etest 24h			[47]	27	75/93	24/48 h	[52]
	25	100	Micro/Etest 24h			[49]	152	97		[51]
*C. tropicalis*	39	90	Micro/Etest 24h			[47]	28	93/89	24/48 h	[52]
	12	100	Micro/Etest 24h			[49]	152	99		[51]
*C. guilliermondii*	11	91	Micro/Etest 24h			[47]				
	19	79	Micro/Etest 24h			[49]				
*C. kefyr*							136	98		[51]
**Anidulafungin**										
*C. albicans*	33	91	Micro/Etest 24h			[48]				
	32	78	Micro/Etest 24h			[49]				
*C. glabrata*	13	69	Micro/Etest 24h			[48]				
	34	91	Micro/Etest 24h			[49]				
*C. krusei*	12	75	Micro/Etest 24h			[48]				
	11	100	Micro/Etest 24h			[49]				
*C. parapsilosis*	57	74	Micro/Etest 24h			[48]				
	25	100	Micro/Etest 24h			[49]				
*C. tropicalis*	15	100	Micro/Etest 24h			[48]				
	12	100	Micro/Etest 24h			[49]				
*C. guilliermondii*	9	78	Micro/Etest 24h			[48]				
	19	95	Micro/Etest 24h			[49]				

Data from [25] performed in AM3 broth instead of RPMI. Data from [47] correspond to 50% inhibition.

**Table 5:** Agreement between Etest and reference techniques (European Committee for Antimicrobial Susceptibility Testing (EUCAST) and Clinical and Laboratory Standards Institute (CLSI)) for *Aspergillus* spp.

Organisms	Number of Isolates	ATF	Reference Technique	Endpoint Used for Comparison	Essential Agreement (EA) with Reference Technique ^a^	Comments	Reference
Aspergillus	123	AMB	CLSI	+/−2 dil	75%–100%	Lowest EA for *A. flavus*, *A. fumigatus*, and *A. nidulans*. Higher EA for Etest at 24h	[60]
Aspergillus	126	AMB	CLSI	+/−2 dil	96%	Higher MICs for Etest	[44]
Aspergillus	154	AMB	CLSI	+/−2 dil	16%–100%	16% for *A. terreus*, 97-100% for other species	[66]
Aspergillus	40	AMB	CLSI	+/−2 dil	60%–100%	50-100% at +/− 1dil	[76]
Aspergillus	63	AMB	CLSI	+/−2 dil	88.5%	Higher EA for Etest at 24h	[67]
Aspergillus	25	AMB	CLSI	+/−2 dil	89.2%	Higher EA for Etest at 24h. higher MICs for Etest	[69]
Aspergillus	107	AMB	CLSI	+/−2 dil	91.7%	Higher EA for Etest at 24h	[61]
Aspergillus	32	AMB	CLSI	+/−2 dil	81%	Higher EA for Etest at 24h	[71]
Aspergillus	48	AMB	CLSI	+/−2 dil	100%	EA at +/− 1 dil: 72% for *A. fumigatus* and 62% for *A. niger* -Etest in MHG	[74]
Aspergillus	87	AMB	CLSI	ND	ND	Only *A. terreus*. Lower MICs for Etest	[59]
Aspergillus	79	AMB	EUCAST	+/−2 dil	79.7%	Only *A. terreus*, CA 88.7%	[78]
Aspergillus	123	ITZ	CLSI	+/−2 dil	83.3%–100%	higher MICs by Etest. Lowest EA for *A. nidulans*	[60]
Aspergillus	29	ITZ	CLSI	+/−1 dil	75.8%	Higher MIC by Etest	[65]
Aspergillus	24	ITZ	CLSI	+/−2 dil	100%	/	[73]
Aspergillus	40	ITZ	CLSI	+/−2 dil	90%–100%	40-100% at +/− 1dil	[76]
Aspergillus	63	ITZ	CLSI	+/−2 dil	67.2%	Higher EA for Etest at 24h. Higher MIC by Etest	[67]
Aspergillus	25	ITZ	CLSI	+/−2 dil	72.5%	Higher EA for Etest at 24h. Higher MIC by Etest	[69]
Aspergillus	376	ITZ	CLSI	+/−2 dil	95.8%	Higher MIC by Etest	[87]
Aspergillus	107	ITZ	CLSI	+/−2 dil	91.8%	Higher EA for Etest at 24h	[61]
Aspergillus	170	ITZ	CLSI	+/−1 dil	93.5%	/	[77]
Aspergillus	32	ITZ	CLSI	+/−2 dil	75%	/	[71]
Aspergillus	50	ITZ	mEUCAST	ND	ND	Only *A. niger*. Lower MIC by Etest	[64]
Aspergillus	79	ITZ	EUCAST	+/−2 dil	73.4%	Only *A. terreus*, higher MIC by Etest. CA 98.7%	[78]
Aspergillus	29	VRZ	CLSI	+/−1 dil	100%	/	[65]
Aspergillus	154	VRZ	CLSI	+/−2 dil	95%–100%	Lower MIC by Etest	[66]
Aspergillus	376	VRZ	CLSI	+/−2 dil	97.6%	Lower MIC by Etest	[87]
Aspergillus	77	VRZ	mCLSI	+/−2 dil	93.5%	Higher EA for Etest at 24 h. Higher MIC by Etest	[75]
Aspergillus	107	VRZ	CLSI	+/−2 dil	96.3%	/	[61]
Aspergillus	32	VRZ	CLSI	+/−2 dil	85%	/	[71]
Aspergillus	48	VRZ	CLSI	+/−2 dil	92%–100%	at +/− 1 dil 36% for *A. fumigatus* and 8% for *A. niger* -Etest in MHG	[74]
Aspergillus	79	VRZ	EUCAST	+/−2 dil	93.7%	Only *A. terreus*, CA 100%	[78]
Aspergillus	126	PSZ	CLSI	+/−2 dil	97%	/	[44]
Aspergillus	29	PSZ	CLSI	+/−1 dil	93%	/	[65]
Aspergillus	154	PSZ	CLSI	+/−2 dil	64%–100%	/	[66]
Aspergillus	55	PSZ	CLSI	+/−2 dil	82%–88%	82% for *A. fumigatus*, 88% for other species. Lower MIC by Etest	[72]
Aspergillus	50	PSZ	CLSI	+/−2 dil	90%–92%	CA 84–88%	[56]
Aspergillus	107	PSZ	CLSI	+/−2 dil	95.3%	/	[61]
Aspergillus	48	PSZ	CLSI	+/−2 dil	100%	at +/− 1 dil 72% for *A. fumigatus* and 85% for *A. niger* -Etest in MHG	[74]
Aspergillus	82	PSZ	CLSI	ND	ND	Lower MIC by Etest	[58]
Aspergillus	140	PSZ	CLSI	ND	ND	CA 99.3%	[70]
Aspergillus	79	PSZ	EUCAST	+/−2 dil	96.2%	Only *A. terreus*, CA 77.2%	[78]
Aspergillus	702	ISA	CLSI	+/−2 dil	96.7%	Lower MIC by Etest	[63]
Aspergillus	79	ISA	EUCAST	+/−2 dil	89%–90%	Only *A. fumigatus*. Lower MIC by Etest	[57]
Aspergillus	79	ISA	EUCAST	+/−2 dil	97.5%	Only *A. terreus*, CA 97.4%	[78]
Aspergillus	154	CAS	CLSI	+/−2 dil	14%–100%	14% for *A. ustus*, 80–100% for other species	[66]
Aspergillus	67	CAS	CLSI	+/−2 dil	79%–83.5%	Higher EA for Etest at 24h	[68]
Aspergillus	169	CAS	CLSI	+/−2 dil	38%–80%	/	[23]
Aspergillus	272	CAS	CLSI	+/−2 dil	61%	26% at +/− 1 dil	[62]
Aspergillus	48	CAS	CLSI	+/−2 dil	100%	at +/− 1 dil 76% for *A. fumigatus* and 62% for *A. niger* -Etest in MHG	[74]
Aspergillus	79	CAS	EUCAST	+/−2 dil	96.2%	Only *A. terreus*	[78]
Aspergillus	154	Mica	CLSI	+/−2 dil	57%–100%	57% for *A. ustus*, 100% for other species	[66]
Aspergillus	67	Mica	CLSI	+/−2 dil	100%	/	[68]
Aspergillus	79	Mica	EUCAST	+/−2 dil	100%	Only *A. terreus*	[78]
Aspergillus	67	ANI	CLSI	+/−2 dil	100%	/	[68]
Aspergillus	48	ANI	CLSI	+/−2 dil	100%	All MIC values < 0.03 µg/mL	[74]
Aspergillus	79	ANI	EUCAST	+/−2 dil	100%	Only *A. terreus*	[78]

^a^, best overall EA value or range by species.

**Table 6:** Agreement between Etest and reference techniques (EUCAST and CLSI) for Mucorales.

Organisms	Number of Isolates	ATF	Reference Technique	Endpoint Used for Comparison	Essential Agreement (EA) with Reference Technique	Comments	Reference
Mucorales	131	AMB	EUCAST	+/− 2 dil	73%	/	[84]
Mucorales	92	AMB	CLSI	+/− 1 dil	96.5%	/	[44]
Mucorales	80	AMB	CLSI	ND	ND	CA 87%	[85]
Mucorales	14	AMB	EUCAST	+/− 2 dil	78.6%	50% at +/− 1 dil	[81]
Mucorales	35	AMB	CLSI	+/− 2 dil	91%	/	[66]
Mucorales	45	AMB	CLSI	+/− 2 dil	70.5%	EA depends on incubation time	[86]
Mucorales	10	AMB	CLSI	+/− 2 dil	90%	90% at +/− 1dil	[76]
Mucorales	21	ITZ	CLSI	+/− 1 dil	80%	/	[65]
Mucorales	6	ITZ	CLSI	+/− 2 dil	83%	/	[73]
Mucorales	45	ITZ	CLSI	+/− 2 dil	70.5%	EA depends on incubation time	[86]
Mucorales	10	ITZ	CLSI	+/− 2 dil	50%	20% at +/− 1dil	[76]
Mucorales	21	VRZ	CLSI	+/− 1 dil	90%	/	[65]
Mucorales	35	VRZ	CLSI	+/− 2 dil	100%	/	[66]
Mucorales	131	PSZ	EUCAST	+/− 2 dil	77%	/	[84]
Mucorales	21	PSZ	CLSI	+/− 1 dil	80%	/	[65]
Mucorales	92	PSZ	CLSI	+/− 1 dil	95.7%	/	[44]
Mucorales	80	PSZ	CLSI	ND	ND	CA 67%	[85]
Mucorales	14	PSZ	EUCAST	+/− 2 dil	100%	78.6% at +/− 1 dil	[81]
Mucorales	35	PSZ	CLSI	+/− 2 dil	94%	/	[66]
Mucorales	45	PSZ	CLSI	+/− 2 dil	88.6%	EA depends on incubation time	[86]
Mucorales	45	ISA	CLSI	+/− 2 dil	84.4%	71.1% at +/− 1 dil	[63]
Mucorales	35	CAS	CLSI	+/− 2 dil	100%	/	[66]
Mucorales	35	Mica	CLSI	+/− 2 dil	100%	/	[66]

**Table 7:** Agreement between Etest and reference techniques (EUCAST and CLSI) for *Fusarium* spp.

Organisms	Number of Isolates	ATF	Reference Technique	Endpoint Used for Comparison	Essential Agreement (EA) with Reference Technique	Comments	Reference
Fusarium	20	AMB	EUCAST	+/−2 dil	95%	80% at +/− 1 dil, CA 100%	[79]
Fusarium	20	AMB	CLSI	+/−2 dil	90%	60% at +/− 1 dil, CA 85%	[79]
Fusarium	7	AMB	EUCAST	+/−2 dil	100%	85.7% at +/− 1 dil	[81]
Fusarium	34	AMB	CLSI	+/−2 dil	94%	/	[66]
Fusarium	54	AMB	EUCAST	+/−2 dil	96%	/	[82]
Fusarium	10	AMB	CLSI	+/−2 dil	70%	10% at +/− 1 dil	[76]
Fusarium	48	AMB	CLSI	+/−2 dil	72.9%	54.2% at +/− 1 dil	[80]
Fusarium	10	AMB	CLSI	+/−2 dil	40–70%	EA depends on incubation time	[60]
Fusarium	7	AMB	CLSI	+/−2 dil	100%	Etest in MHG	[74]
Fusarium	13	ITZ	CLSI	+/−2 dil	100%	/	[73]
Fusarium	54	ITZ	EUCAST	+/−2 dil	100%	/	[82]
Fusarium	10	ITZ	CLSI	+/−2 dil	90%	90% at +/− 1 dil	[76]
Fusarium	10	ITZ	CLSI	+/−2 dil	100%	/	[60]
Fusarium	5	ITZ	CLSI	+/− 1 dil	80%	/	[65]
Fusarium	20	VRZ	EUCAST	+/−2 dil	95%	75% at +/− 1 dil, CA 95%	[79]
Fusarium	20	VRZ	CLSI	+/−2 dil	95%	80% at +/− 1 dil, CA 95%	[79]
Fusarium	34	VRZ	CLSI	+/−2 dil	100%	/	[66]
Fusarium	54	VRZ	EUCAST	+/−2 dil	100%	/	[82]
Fusarium	48	VRZ	CLSI	+/−2 dil	91.7%	62.5% at +/− 1 dil	[80]
Fusarium	5	VRZ	CLSI	+/− 1 dil	80%	/	[65]
Fusarium	7	VRZ	CLSI	+/−2 dil	100%	Etest in MHG	[74]
Fusarium	20	PSZ	EUCAST	+/−2 dil	100%	45% at +/− 1 dil, CA 90%	[79]
Fusarium	20	PSZ	CLSI	+/−2 dil	85%	70% at +/− 1 dil, CA 90%	[79]
Fusarium	7	PSZ	EUCAST	+/−2 dil	100%	100% at +/− 1 dil	[81]
Fusarium	34	PSZ	CLSI	+/−2 dil	100%	/	[66]
Fusarium	54	PSZ	EUCAST	+/−2 dil	96%	/	[82]
Fusarium	5	PSZ	CLSI	+/− 1 dil	60%	/	[65]
Fusarium	7	PSZ	CLSI	+/−2 dil	100%	Etest in MHG	[74]
Fusarium	20	ISA	CLSI	+/−2 dil	85%	65% at +/− 1 dil	[63]
Fusarium	34	CAS	CLSI	+/−2 dil	100%	/	[66]
Fusarium	10	CAS	CLSI	+/−2 dil	100%	All isolates R	[68]
Fusarium	7	CAS	CLSI	+/−2 dil	100%	Etest in MHG, all isolates R	[74]
Fusarium	34	Mica	CLSI	+/−2 dil	100%	/	[66]
Fusarium	10	Mica	CLSI	+/−2 dil	100%	All isolates R	[68]
Fusarium	10	ANI	CLSI	+/−2 dil	100%	All isolates R	[68]
Fusarium	7	ANI	CLSI	+/−2 dil	100%	Etest in MHG, all isolates R	[74]

**Table 8:** Agreement between Etest and reference techniques (EUCAST and CLSI) for *Scedosporium* spp.

Organisms	Number of Isolates	ATF	Reference Technique	Endpoint Used for Comparison	Essential Agreement (EA) with Reference Technique	Comments	Reference
Scedosporium	10	AMB	CLSI	+/−2 dil	100%	/	[60]
Scedosporium	15	AMB	CLSI	+/−2 dil	20% Sa / 20% Sp	20% Sa / 20% Sp at +/− 1 dil	[76]
Scedosporium	25	AMB	CLSI	+/−2 dil	80% Sa / 100% Sp	/	[66]
Scedosporium	10	ITZ	CLSI	+/−2 dil	100%	/	[60]
Scedosporium	15	ITZ	CLSI	+/−2 dil	60% Sa / 100% Sp	50% Sa / 100% Sp at +/− 1 dil	[76]
Scedosporium	5	ITZ	CLSI	+/−2 dil	100%	/	[73]
Scedosporium	25	VRZ	CLSI	+/−2 dil	90% Sa / 100% Sp	/	[66]
Scedosporium	31	VRZ	CLSI	+/−2 dil	93.5%	87.1% at +/− 1 dil; CA 93.6%	[83]
Scedosporium	25	PSZ	CLSI	+/−2 dil	90% Sa / 100% Sp	/	[66]
Scedosporium	22	ISA	CLSI	+/−2 dil	18.7% Sa / 100% Sp	6.25% Sa / 100% Sp at +/− 1 dil	[63]
Scedosporium	25	CAS	CLSI	+/−2 dil	63% Sa / 100% Sp	/	[66]
Scedosporium	25	Mica	CLSI	+/−2 dil	37% Sa / 100% Sp	/	[66]

Sa, *Scedosporium apiospermum* and Sp, *Scedosporium prolificans*.

**Table 9:** Specific Etest ECVs for amphotericin B and azoles as compared to CLSI and EUCAST for *Candida* spp. and *Aspergillus* spp.

Species	ECV (µg/ml) for														
	Amphotericin B			Fluconazole			Itraconazole			Voriconazole			Posaconazole		
	Etest	CLSI	EUCAST	Etest	CLSI	EUCAST	Etest	CLSI	EUCAST	Etest	CLSI	EUCAST	Etest	CLSI	EUCAST
*C. albicans*	1 ^a^	2 ^a^	1 ^c^	1 ^b^	0.5 ^e^	1 ^c^	0.25 ^e^	NA	0.064 ^c^	0.03 ^e^	0.03 ^e^	0.125 ^c^	0.12 ^e^	0.06 ^e^	NA
*C. glabrata*	2 ^a^	2 ^a^	1 ^c^	64 ^e^	8 ^e^	32 ^c^	8 ^e^	4 ^e^	2.0 ^c^	2 ^e^	0.25 ^e^	1.0 ^c^	NA	1 ^e^	NA
*C. krusei*	4 ^a^	2 ^a^	1 ^c^	NA	32 ^e^	128 ^c^	2 ^e^	1 ^e^	1.0 ^c^	2 ^e^	0.5 ^e^	1.0 ^c^	NA	0.5 ^e^	NA
*C. parapsilosis*	2 ^a^	2 ^a^	1 ^c^	4 ^e^	1 ^e^	2 ^c^	NA	NA	0.125 ^c^	0.25 ^e^	0.03 ^e^	0.125 ^c^	0.12 ^e^	0.25 ^e^	NA
*C. tropicalis*	2 ^a^	2 ^a^	1 ^c^	4 ^e^	1 ^e^	2 ^c^	0.5 ^e^	0.5 ^e^	0.125 ^c^	0.5 ^e^	0.12 ^e^	0.125 ^c^	0.12 ^e^	0.12 ^e^	NA
*C. dubliniensis*	0.5 ^a,d^	NA	NA	NA	0.5 ^e^	NA	NA	NA	0.064 ^c^	NA	0.03 ^e^	NA	NA	0.25 ^e^	NA
*C. kefyr*	2 ^b^	NA	NA	1 ^b^	NA	NA	NA	NA	NA	0.03 ^b^	NA	NA	NA	NA	NA
*C. lusitaniae*	1 ^b^	NA	NA	1 ^b^	1 ^e^	NA	NA	0.5 ^e^	0.125 ^c^	0.03 ^b^	0.06 ^e^	0.064 ^c^	NA	0.06 ^e^	NA
*C. guilliermondii*	1 ^b^	NA	NA	4 ^b^	8 ^e^	16 ^c^	NA	NA	2.0 ^c^	0.125 ^b^	0.12 ^e^	0.25 ^c^	NA	0.5 ^e^	NA
*A. fumigatus*	2 ^a^	2 ^a^	1 ^a^	NA	NA	NA	2 ^e^	1 ^e^	1.0 ^c^	0.5 ^e^	1 ^e^	1.0 ^c^	0.25 ^e^	0.25 ^e^	NA
*A. flavus*	8 ^a^	4 ^a^	4 ^a^	NA	NA	NA	1 ^e^	1 ^e^	1.0 ^c^	0.5 ^e^	2 ^e^	2.0 ^c^	0.5 ^e^	0.5 ^e^	NA
*A. niger*	2 ^a^	2 ^a^	1 ^a^	NA	NA	NA	4 ^e^	4 ^e^	4.0 ^c^	1 ^e^	2 ^e^	2.0 ^c^	0.5 ^e^	2 ^e^	NA
*A. terreus*	16 ^a^	4 ^a^	4 ^a^	NA	NA	NA	NA	2 ^e^	0.5 ^c^	NA	2 ^e^	2.0 ^c^	0.25 ^e^	1 ^e^	NA
*A. nidulans*	NA	NA	NA	NA	NA	NA	NA	NA	1 ^c^	NA	NA	NA	NA	NA	NA

^a^, data from [89]; ^b^, data from [109]; ^c^, data from the EUCAST database (https://mic.eucast.org/Eucast2/); ^d^, value after normalization of data; and ^e^, data from [101].

**Table 10:** Specific Etest ECVs for echinocandins as compared to CLSI and EUCAST for *Candida* spp. and *A. fumigatus.*

Species	ECV (µg/mL) for								
	Caspofungin			Micafungin			Anidulafungin		
	Etest	CLSI	EUCAST	Etest	CLSI	EUCAST	Etest	CLSI	EUCAST
*C. albicans*	0.5 ^a^	NA	NA	0.03 ^a^	0.03 ^a^	0.015 ^c^	0.016 ^a^	0.12 ^a^	0.03 ^c^
*C. glabrata*	1 ^a^	NA	NA	0.03 ^a^	0.03 ^a^	0.03 ^c^	0.03 ^a^	0.12 ^a^	0.06 ^c^
*C. krusei*	1 ^a^	NA	NA	0.25 ^a^	0.25 ^a^	0.25 ^c^	0.06 ^a^	0.25 ^a^	0.06 ^c^
*C. parapsilosis*	4 ^a^	NA	NA	2 ^a^	4 ^a^	2 ^c^	8 ^a,d^	8 ^a^	4 ^c^
*C. tropicalis*	1 ^a,d^	NA	NA	0.12 ^a,d^	0.06 ^a^	0.06 ^c^	0.03 ^a^	0.06 ^a^	0.06 ^c^
*C. kefyr*	0,25 ^b^	NA	NA	0.25 ^b^	NA	NA	NA	NA	NA
*C. lusitaniae*	1 ^b^	NA	NA	NA	NA	NA	NA	NA	NA
*C. guilliermondii*	2 ^b^	NA	NA	NA	NA	NA	NA	NA	NA
*A. fumigatus*	0.125 ^d^	NA	NA	0.016 ^b,d^	NA	NA	NA	NA	NA

^a^, data from [89]; ^b^, data from [109]; ^c^, data from the EUCAST database (https://mic.eucast.org/Eucast2/); and ^d^, value after normalization of data.
